# Inhibition of mitochondrial autophagy protects donor lungs for lung transplantation against ischaemia‐reperfusion injury in rats via the mTOR pathway

**DOI:** 10.1111/jcmm.14177

**Published:** 2019-03-19

**Authors:** Wei‐Cheng Liu, Shi‐Biao Chen, Sheng Liu, Xiang Ling, Qi‐Rong Xu, Ben‐Tong Yu, Jian Tang

**Affiliations:** ^1^ Department of Anesthesiology the First Affiliated Hospital of Nanchang University Nanchang P.R. China; ^2^ Department of Thoracic Surgery the First Affiliated Hospital of Nanchang University Nanchang P.R. China

**Keywords:** hypoxia‐reoxygenation, lung injury, lung ischaemia‐reperfusion, mitochondrial autophagy, mTOR pathway, pulmonary microvascular endothelial cells

## Abstract

Impaired mitochondrial function is a key factor attributing to lung ischaemia‐reperfusion (IR) injury, which contributes to major post‐transplant complications. Thus, the current study was performed to investigate the role of mitochondrial autophagy in lung I/R injury and the involvement of the mTOR pathway. We established rat models of orthotopic left lung transplantation to investigate the role of mitochondrial autophagy in I/R injury following lung transplantation. Next, we treated the donor lungs with 3‐MA and Rapamycin to evaluate mitochondrial autophagy, lung function and cell apoptosis with different time intervals of cold ischaemia preservation and reperfusion. In addition, mitochondrial autophagy, and cell proliferation and apoptosis of pulmonary microvascular endothelial cells (PMVECs) exposed to hypoxia‐reoxygenation (H/R) were monitored after 3‐MA administration or Rapamycin treatment. The cell apoptosis could be inhibited by mitochondrial autophagy at the beginning of lung ischaemia, but was rendered out of control when mitochondrial autophagy reached normal levels. After I/R of donor lung, the mitochondrial autophagy was increased until 6 hours after reperfusion and then gradually decreased. The elevation of mitochondrial autophagy was accompanied by promoted apoptosis, aggravated lung injury and deteriorated lung function. Moreover, the suppression of mitochondrial autophagy by 3‐MA inhibited cell apoptosis of donor lung to alleviate I/R‐induced lung injury as well as inhibited H/R‐induced PMVEC apoptosis, and enhanced its proliferation. Finally, mTOR pathway participated in I/R‐ and H/R‐mediated mitochondrial autophagy in regulation of cell apoptosis. Inhibition of I/R‐induced mitochondrial autophagy alleviated lung injury via the mTOR pathway, suggesting a potential therapeutic strategy for lung I/R injury.

## 1 INTRODUCTION

Ischaemia and following reperfusion are commonly occurring processes in various medical situations, including major surgical procedures and organ transplantation, often resulting in devastating consequences, such as tissue injury.^1^ Lung ischaemia‐reperfusion (I/R) injury, a common complication in lung transplantation, remains to be the leading cause of death after lung transplantation.^2^ There are multiple characteristics of lung I/R injury, including infiltration of inflammatory cells, interstitial oedema and disruption of respiratory membranes.^3^ It is reported that I/R‐induced lung injury is related to severe haemorrhagic shock, pulmonary thromboendarterectomy, aortic surgery and open heart surgery.^4^ The development of lung I/R injury leads to prolonged duration of mechanical ventilation, increased attendance in intensive care unit, duration of hospital stays and high morbidity and mortality.^5^ In recent years, extensive investigations have explored the impacts of neutrophils and other inflammatory mediators on lung I/R injury, however, there seems to be a lack of satisfactory therapy regimens for pulmonary protection, and the understanding of the specific mechanisms of lung I/R injury remains unclear.^6^, ^7^

Autophagy is considered as an evolutionary highly conversed process among all eukaryotes ranging from yeast to mammals.^8^ It is known that mitochondrial autophagy is often caused by exposure to hypoxic environments, and autophagic cellular damage contributes to I/R injury during the process of I/R.^9^ Mitochondrial autophagy participates in various aspects of tissue homeostasis and exerts an essentially physiological effect on eukaryotic cells.^10^, ^11^ In addition, mitochondrial autophagy not only plays a role in cell survival, but also affects cell death.^12^ Moreover, a recent study manifested the role of mitochondrial autophagy in the maintenance of mitochondrial homeostasis and quality control, cellular development and differentiation.^13^ With this aforementioned dual role, mitochondrial autophagy is also involved in the pathogenesis of various diseases, including cancer, heart disease, neurodegeneration and infections.^14^ For instance, selective mitochondrial autophagy in conjunction with macroautophagy alleviates the ability of chondrogenesis in adipose stem cells (ASCs) of equine metabolic syndrome (EMS).^15^ Similarly, a more recent study reported that selective mitochondrial autophagy induced by endoplasmic reticulum and mitochondrial stress exerts a protective role in ASC_EMS_ against cell death.^16^ Rapamycin, an immunosuppressant, was identified to promote mitochondrial autophagy by suppressing the mammalian target of rapamycin (mTOR) pathway.^17^ Additionally, serine‐threonine kinase mTOR is regarded as an intracellular sensor of energy metabolism and stress, which is associated with cellular growth and metabolism.^18^ Wang et al have confirmed that the mTOR pathway plays distinct roles in the process of inflammation and acute lung injury (ALI).^19^ The mTOR pathway affects I/R injury in kidneys by regulation of mitochondrial autophagy.^20^ Moreover, the mTOR pathway was also reported to exert effects on ALI in mice by mediating mitochondrial autophagy of lung tissue cells.^21^ Therefore, the current study was performed aiming to elucidate the mechanism by which mitochondrial autophagy exerts its effect on lung I/R injury by regulating the mTOR pathway.

## 2 MATERIALS AND METHODS

### 2.1 Ethical statements

All animal experimentation in the current study was approved by the Animal Ethics Committee of the First Affiliated Hospital of Nanchang University. Maximal efforts were made to minimize the suffering of the included animals.

### 2.2 Experimental animals

A total of 180 Sprague‐Dawley (SD) rats (weighing 200‐250 g) raised under specific pathogen‐free (SPF) conditions were included in the current study. The included rats were classified as donors and recipients. The number of recipient mice was slightly larger than that of the donor mice, and differences in weight between the donor and recipient were no more than 25 g. All rats underwent left lung transplantation. Prior to the surgery, the recipient mice were fasted for 8 hours but allowed free access to water, while free access to food and water was resumed after surgery.

### 2.3 Model establishment

All rats were treated with intraperitoneal anaesthesia. Next, an incision was made in the median chest and abdomen of the rats. After the chest cavity was opened, the root of the lung artery was treated with perfusion fluid, and the inferior vena cava was incised open. When the lungs became completely pale, the cardiopulmonary specimen block was extracted and placed on cool gauze. Next, the lung tissues were freely dissected, followed by vessel cannulation and bronchus clipping. The lung tissues from the donor group were preserved in cold perfusion fluid. One group of rats underwent ischaemia treatment, and the other group of rats were treated with donor lung reperfusion as follows: rats from the donor group were anaesthetized. After the anastomosis of the pulmonary artery, trachea, vein of donor rats and hilar structure of recipient rats, the pulmonary artery was opened. Subsequently, the specimens were collected for further experimentation.

### 2.4 Lung specimen treatment

1. Cold ischaemia preservation (N = 6): The lung tissues from donor rats were preserved in the mitochondrial autophagy inhibitor 3‐MA, Rapamycin (mTOR pathway inhibitor) and ordinary perfusion fluid as the 3‐MA group, Rapamycin group and control group respectively. Next, cold ischaemia preservation was conducted for different time intervals (1, 3, 6, 9 and 12 hours) respectively.
2. Reperfusion (N = 6): The lung tissues preserved in the 3‐MA, Rapamycin and control groups were transplanted after cold ischaemia preservation for different time intervals (1, 3, 6, 9 and 12 hours) respectively. After reperfusion for 3 hours, the lung function was assessed.

Next, specimens were prepared for electron microscopy observation. After the thorax of rats was exposed, the inferior lobe of the right lung was quickly removed and rinsed in pre‐cooled PBS. Then the lung tissues were cut into pieces, and fixed in 2.5% glutaraldehyde. The tissue sections were collected after shaking several times and then observed under an electron microscope.

### 2.5 Isolation, culture and identification of pulmonary microvascular endothelial cells (PMVECs)

Rats were intraperitoneally anaesthetized with 3% pentobarbital sodium (0.1 mL/100 g), and sterile PBS was perfused into the right ventricle. When the lung tissues became completely pale, the cardiopulmonary tissues were quickly extracted, and pleura lung tissues were taken. After being rinsed, the tissues were sliced into 1‐3 mm^3^ blocks, and evenly inoculated into culture bottles of 25 cm^2^. The blocks were added with 1.5 mL DMEM complete culture medium (containing 100 kU/L penicillin, 100 kU/L streptomycin, 90 kU/L heparin, 50 μg/L serum, VEGF and 20% foetal bovine serum (FBS) with immersion blocks avoiding tissue mass floating. Next, the blocks were cultured in an incubator, and the culture medium was replaced every day to remove haemocytes. After adhering to the wall for 60 hours, the tissue blocks were carefully removed, and the culture medium was changed for subsequent incubation. The medium was changed once every 3 days until the monolayer cells covered about 90% of the area. The cells were treated with 0.25% trypsin and passaged. The cells of the third generation were used for subsequent experimentation. Immunofluorescence was adopted to determine the expression of marker CD31 to identify PMVECs.

### 2.6 Hypoxia‐reoxygenation (H/R) model establishment

The cells were assigned into normal, hypoxia, and H/R groups, and cultured in a hypoxic incubator containing 50% N_2_, 5% CO_2_, 45% air for different time intervals (3, 6 and 9 hours). The cells in the H/R group were cultured in a hypoxic incubator for different time intervals (3, 6 and 9 hours), and transferred to the conventional incubator (5% CO_2_, 95% air) for 3 hours. All groups were intervened by 3‐MA or Rapamycin 1 hour prior to the experiment. All experiments were independently repeated three times.

### 2.7 Lung function evaluation

The injury of lung tissues was detected and the lung function was assessed. Cold ischaemia specimens were directly preserved in perfusion fluid, and the specimens were taken out for detection when the scheduled time‐point was reached. The recipient rats were treated with trachea cannulation under general anaesthesia, followed by respiratory ventilation. The corresponding instruments were employed to monitor the indicators of lung functioning. The donor lung specimens were obtained through the original incision, and stored in a refrigerator with deep hypothermia. Peak inspiratory pressure (PIP) detection was performed as follows: the trachea cannula was connected to the three‐limb tubes, which were connected to the breathing pressure assay instrument; the peak value of the pressure gauge was read and recorded. Blood gas analysis (PO_2_) for pulmonary veins was performed as follows: after the chest was opened, the left pulmonary vein was fully exposed; venous blood samples were collected from the roots of the pulmonary vein using a heparin catheter; blood gas analysis was carried out and the data were recorded.

### 2.8 Wet/Dry (W/D) ratio of lung tissues

The lung tissue blocks (0.5 g) were collected and washed to remove superficial blood. The excess liquid on the surface was absorbed using absorbent paper, and the tissue blocks were weighed, which was referred to as the wet weight. The tissue blocks were dried in a thermostat oven for 2 hours and weighed again, which was referred to as the dry weight. The ratio of the two values was used to determine the W/D ratio.

### 2.9 Terminal deoxynucleotidyl transferase‐mediated dUTP‐biotin nick end labelling (TUNEL) assay

Apoptosis in lung tissues was detected using the TUNEL assay with an in situ apoptosis detection kit by staining the samples with streptavidin‐peroxidase (SP). An optical microscope was employed for observation of the stained samples. The experimental data and results were recorded in detail. A total of five high‐power fields with the greatest number of positive cells (cell nuclei stained brown) were selected from the TUNEL positive sections from each group (× 400), and the ratio of positive cells out of 500 cells was calculated, which was recorded as the apoptotic index (AI).

### 2.10 Immunofluorescence LC‐II/LAMP double staining

The lung tissue blocks were placed in fixation liquid (10% formalin and Bouin fixation liquid), dehydrated using high concentration gradient alcohol and cleared with xylene. The cleared blocks were immersed in dissolved paraffin, and kept warm in wax dissolution box. When paraffin was completely immersed in tissue blocks, the tissue blocks were embedded. Next, the tissue blocks were sliced into 5‐8 μm thick sections, ironed out in hot water and then attached to glass slides. Subsequently, the sections were dried in a thermostat box at 45℃. The paraffin‐embedded sections were dewaxed into the water. The dewaxed sections were successively immersed in xylene I and xylene II for 15 minutes, respectively, and then successively immersed in anhydrous alcohol I, anhydrous alcohol II, 95% alcohol, 90% alcohol, 80% alcohol, 70% alcohol, each for 5 minutes, and finally washed with distilled water. The sections were placed in a repairing container containing the citric acid (PH6.0)/ethylenediamine tetra‐acetic acid (EDTA) antigen retrieval buffer solution (PH8.0/PH9.0), and placed in a microwave oven for antigen retrieval. After being allowed to cool down at room temperature, the glass slides were placed in PBS (PH7.4), and shaken three times on the shaker for decolourization (5 minutes per time). After the glass slides were dried, a histochemical pen was used to draw a circle around the sections. Subsequently, 3% bovine serum albumin (BSA) was dropped into the circle and sealed at room temperature for 30 minutes. After removal of BSA, the primary antibody (dilution ratio of 1:50, ab48394, LC3B: 1 µg/mL) was added to cover the section, and incubated at 4℃ overnight. The sections were rinsed with PBS thrice (5 minutes per time). After the sections were dried, the secondary antibody with the corresponding species of the primary antibody was added to the circle, and incubated avoiding exposure to light at room temperature for 1 hour. After being rinsed with PBS, the sections were shaken three times for decolourization (5 minutes per time), and the glass slides were placed in PBS. After the sections were dried, the 4', 6‐diamidino‐2‐phenylindole (DAPI) was added into the circle and incubated avoiding light exposure at room temperature for 10 minutes. The sections were rinsed with PBS, and the sections were mounted in corresponding antifade mounting medium. The OLYMPUS inverted fluorescence microscope was employed to observe the sections, under which images were captured.

### 2.11 Flow cytometry

Cells in the logarithmic phase of growth were seeded in a 6‐well plate (cell density of 1 × 10^5^ cells/mL). Next, the cells were treated corresponding to their grouping, and three replicate wells were set for each group. After 48 hours, the cells were collected, rinsed with PBS twice, centrifuged at 44 *g* for 5 minutes and collected again. After removal of the supernatant, there was about 50 μL of PBS remaining. The cell samples were added with 500 μL binding buffer and resuspended in each tube. Then the samples were mixed with 5 μL AnnexinV‐fluorescein isothiocyanate (FITC) and 5 μL Propidium Iodide (PI), followed by incubation avoiding light exposure at room temperature for 10 minutes and two PBS rinses (3 minutes per time). Next, 5 minutes prior to detection on a flow cytometer (Cube6, Partec, Germany), the samples were added with 5 µL PI on an ice bath avoiding light exposure for 5 minutes. The FITC was detected at the wavelength of 480 and 530 nm and PI was detected at the wavelength of more than 575 nm.

### 2.12 Cell counting kit‐8 (CCK‐8) assay

The cell suspension was inoculated in a 96‐well plate (100 μL/well), which was pre‐cultured in a humidified incubator with 5% CO_2_ in air at 37℃. Next, the CCK‐8 solution (40203ES60, Shanghai YEASEN Biotechnology Co., Ltd., Shanghai, China) was added (10 μL/well), and the plate was cultured in an incubator for 1‐4 hours. Additionally, a microplate reader (Molecular Devices, Sunnyvale, CA, USA) was employed to detect the optical density (OD) values using an excitation wavelength of 450 nm.

### 2.13 JC‐1 staining

The JC‐1 fluorescent probe detection mitochondrial transmembrane potential (MTP) kit (M8650, Solarbio Technology Co., Ltd., Beijing, China) was employed in this assay. When the MTP was higher, JC‐1 was gathered in the mitochondrial matrix to form polymers, which produced red fluorescence. When the MTP was lower, JC‐1 was a monomer, producing green fluorescence. The fluorescent enzyme‐labelled instrument was adopted to detect JC‐1 polymer flow cytometry and immunofluorescence (excitation wavelength and emission wavelength were 525 and 590 nm respectively) and JC‐1 monomer (excitation wavelength and emission wavelength were 490 and 530 nm respectively). The MTP level was presented as the ratio of JC‐1 polymer to JC‐1 monomer to represent the mitochondrial permeability transition pore (MPTP) opening and evaluate mitochondrial damage.

### 2.14 Western blot analysis

Total protein content was extracted from cells and tissues, and protein concentration was determined using a bicinchoninic acid (BCA) kit (P0009, Beyotime Biotechnology Co., Ltd., Shanghai, China). The obtained protein samples were mixed with the loading buffer, and heated at 95℃ for 10 minutes. The protein samples (40 μg/well) were separated on 10% polyacrylamide gel (EC62755BOX, Invitrogen Inc, Carlsbad, CA, USA). The separated proteins were transferred to a polyvinylidene fluoride (PVDF) membrane using the wet‐transfer method, and then blocked with 5% BSA at room temperature for 1 hour. Next, the membrane was incubated with primary rabbit antibodies against rat LC3‐II/I (dilution ratio of 1:3000, ab51520), Beclin‐1 (dilution ratio of 1:2000, ab207612), PTEN‐induced kinase 1 (PINK1) (dilution ratio of 1:1000, ab23707), Parkin (5 µg/mL, ab77924), mTOR (dilution ratio of 1:2000, ab2732), p‐mTOR (dilution ratio of 1:2000, ab109268) and glyceraldehyde‐3‐phosphate dehydrogenase (GAPDH) (dilution ratio of 1:2000, ab8245) at 4℃ overnight. Subsequently, the membrane was rinsed with PBS at room temperature thrice (5 minutes per time), and rinsed with Tris‐buffered saline Tween‐20 (TBST) thrice (10 minutes per time). The secondary goat antibody against rabbit immunoglobulin G (IgG) (dilution ratio of 1:2000, ab6721, Abcam, Inc, MA, USA) labelled by horseradish peroxidase (HRP) was incubated with the membrane at room temperature for 1 hour. After being rinsed with PBS thrice (10 minutes per time), the membrane was incubated on a shaking table for 2 hour, followed with rinsing with TBST thrice (10 minutes per time). The membrane was then immersed in electrogenerated chemiluminescence (ECL) reaction solution (WBKLS0100, Millipore Corp., Bedford, MA, USA) for signal developing, and photographed using the SmartView Pro 2000 gel imaging system (UVCI‐2100, Major Science, Saratoga, CA, USA). Additionally, the Gel‐Pro Analyzer 4.0 (Media Cybernetics, Inc, Bethesda, MD, USA) was employed to analyse the gray values of the acquired images. The experiment was repeated three times, and was also applied for subsequent cell experimentation.

### 2.15 Statistical analysis

Statistical analyses were performed with the SPSS 21.0 software (IBM Corp. Armonk, NY, USA). Measurement data were expressed as a mean ± SD. Comparisons between two groups were analysed using the unpaired *t* test, and comparisons among multiple groups were analysed using one‐way ANOVA. A value of *P* < 0.05 was considered to be statistically significant.

## 3 RESULTS

### 3.1 Cold ischaemia‐induced mitochondrial autophagy affects cell apoptosis and lung injury

**Figure 1:**
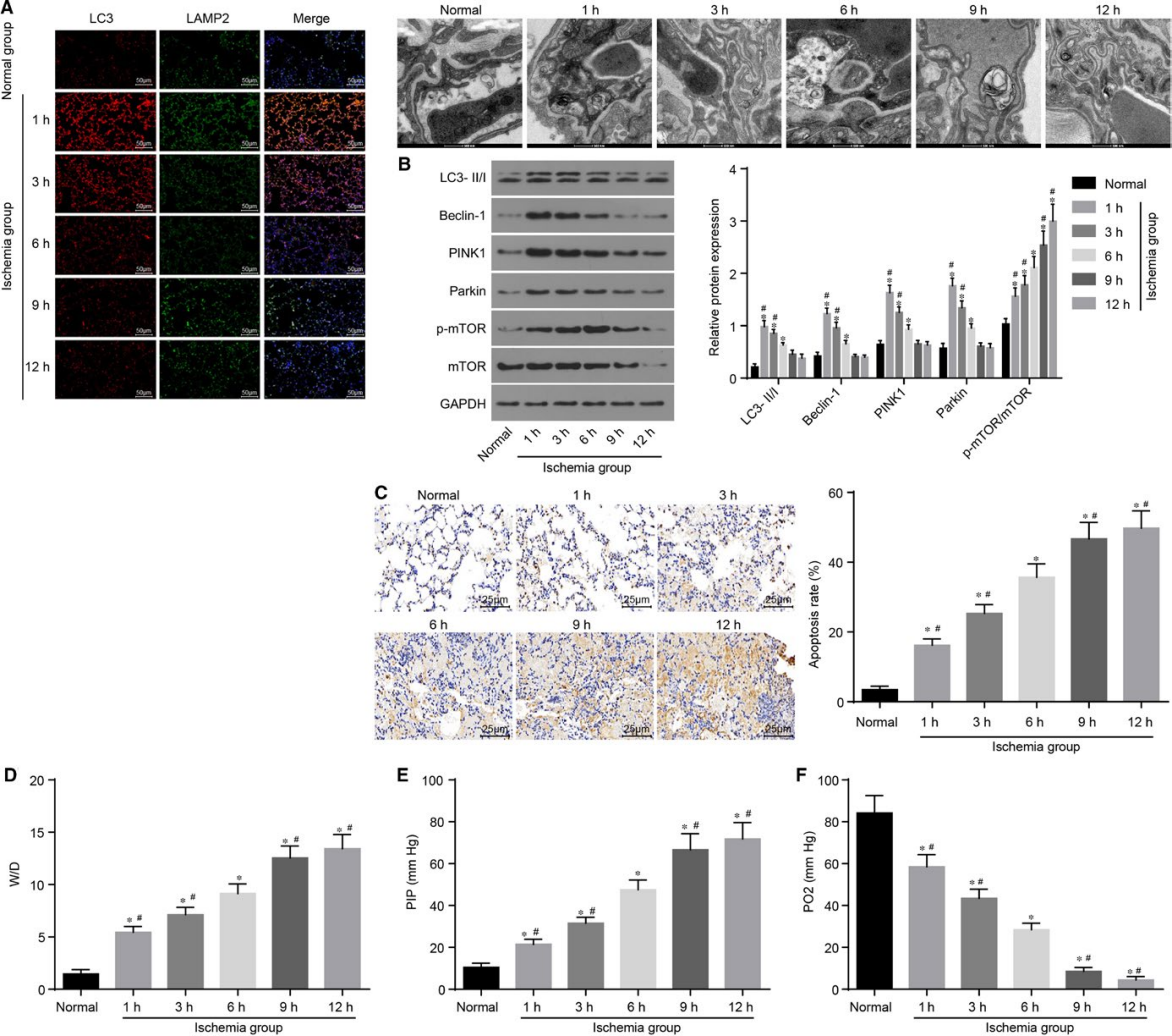
Cold ischaemia at different time intervals affects mitochondrial autophagy and cell apoptosis in lung tissues. A, immunofluorescence LC‐II/LAMP2 double staining (× 200) and transmission electron microscope images shows that fluorescence intensities of LC‐II and LAMP2 are higher in the ischaemia groups than the normal group; B, the extent of mTOR phosphorylation and ratio of p‐mTOR/mTOR expression are increased with the reduction of mitochondrial autophagy in the ischaemia groups; C, TUNEL staining (× 400) shows that cell apoptotic rate is negatively related to mitochondrial autophagy; D, W/D ratio of lung tissues is evidently elevated in the ischaemia groups; E, PIP value of lung tissues is elevated significantly in the ischaemia groups; F, PO_2_ value of lung tissues is reduced dramatically in the ischaemia groups; *P < 0.05, vs the normal group; ^#^ *P* < 0.05, vs the cold ischaemia at 6 h; mTOR, mammalian target of rapamycin; LC‐II, lateral compression type 2; LC3‐II/I, lateral compression type 3‐II/I; LAMP2, lysosome‐associated membrane protein 2; PIP, peak inspiratory pressure; PINK1, PTEN‐induced kinase 1; W/D, wet/dry. All data are expressed as mean ± SD; comparisons among multiple groups are analysed by the one‐way ANOVA; all experiments are repeated three times

Initially, the current study detected lung tissue injury, mitochondrial autophagy and cell apoptosis by changing the time of cold ischaemia in order to study the influence of cold ischaemia at different time intervals on lung injury, mitochondrial autophagy and cell apoptosis. Additionally, immunofluorescence LC‐II/LAMP2 double staining and transmission electron microscope were employed to detect the mitochondrial autophagy of donor lungs. Compared with the normal group, the ischaemia group presented with significantly increased fluorescence intensity of mitochondrial autophagy markers LC‐II and LAMP2, and as the cold ischaemia time extended, the mitochondrial autophagy gradually decreased to normal levels after 6 hours (all *P* < 0.05) (Figure 1A); the protein levels of LC3‐II/I, Beclin‐1, PINK1 and Parkin related to mitochondrial autophagy were increased initially, and then decreased; at different time intervals, along with decreased levels of mitochondrial autophagy, and increased expression ratio of p‐mTOR/mTOR (Figure 1B) (all *P* < 0.05). Furthermore, TUNEL staining was performed to detect cell apoptosis in all groups. Compared with the normal group, the ischaemia group presented with increased cell apoptosis, in addition to decreased mitochondrial autophagy activity; after 6 hours when mitochondrial autophagy attained normal levels, cell apoptosis was found to be accelerated (Figure 1C) (all *P* < 0.05). The W/D and PIP values of lung tissues in each group were gradually elevated, while PO_2_ was gradually reduced (Figure 1D‐F) (all *P* < 0.05). The above findings demonstrated that mitochondrial autophagy was gradually decreased as the cold ischaemic time of donor lung was prolonged. Initially, the mitochondrial autophagy at high levels was able to inhibit cell apoptosis to protect against lung injury. However, after 6 hours, mitochondrial autophagy at normal levels could not regulate cell apoptosis, and consequently, apoptosis was sharply accelerated.

**Figure 2:**
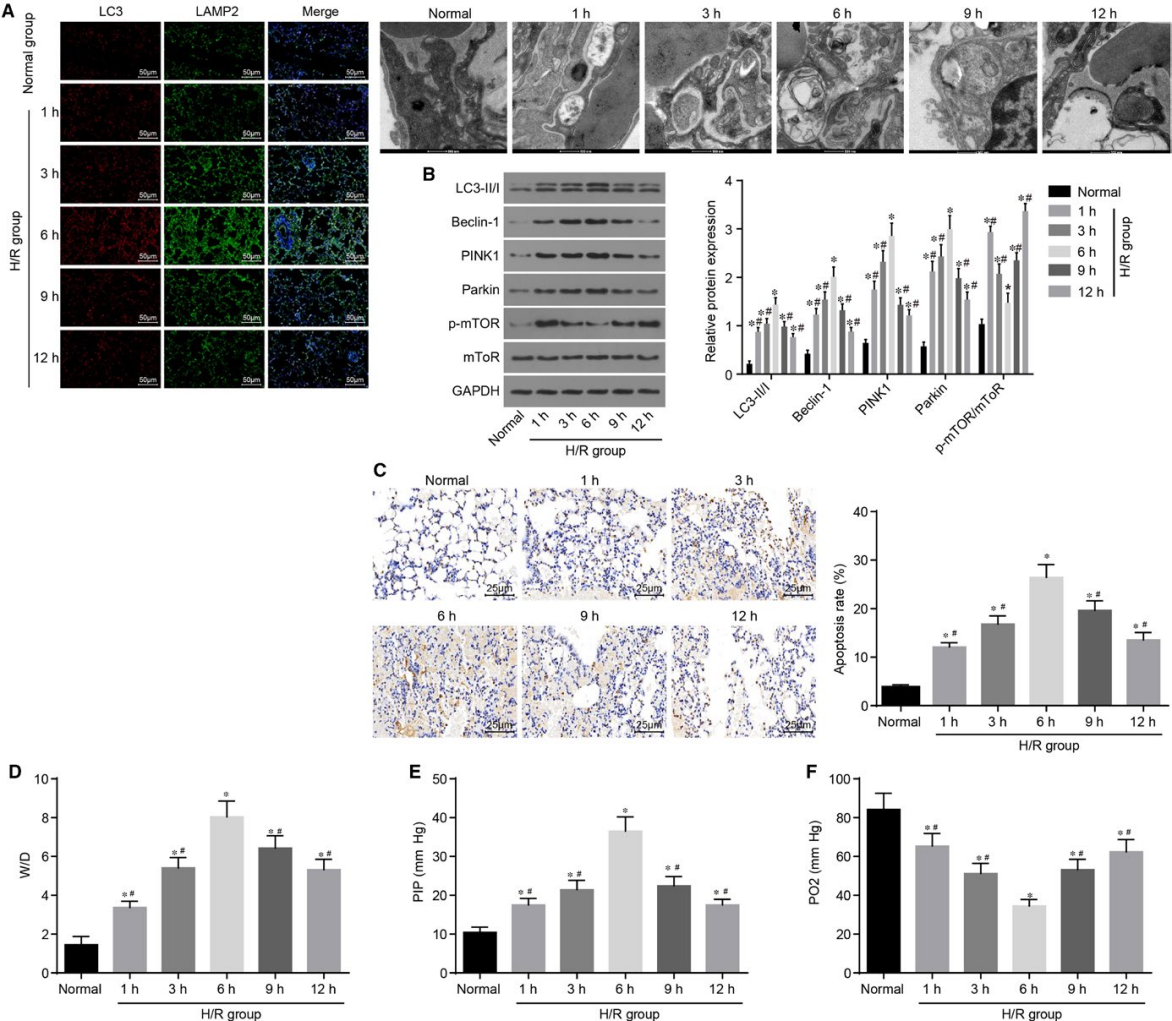
Different time intervals of I/R affect mitochondrial autophagy and apoptosis levels of donor lung tissues. A, immunofluorescence LC‐II/LAMP2 double staining and transmission electron microscope images (× 200) show that fluorescence intensities of mitochondrial autophagy markers LC‐II and LAMP2 are significantly higher at each time point after I/R than those under normal condition; B, protein levels of LC3‐II/I, Beclin‐1, PINK1 and Parkin markedly increase and reach peak values at 6 h after I/R, while the extent of mTOR phosphorylation and p‐mTOR/mTOR exhibit opposite tendencies; C, TUNEL staining (× 400) shows that cell apoptotic rate markedly increases and reaches peak values at 6 h after I/R; D, W/D ratio of lung tissues is elevated and reaches the peak value at 6 h after I/R; E, PIP value of lung tissues is elevated and reaches the peak value after I/R; F, PO_2_ value of lung tissues is reduced and reaches the bottom value after I/R; *P < 0.05, vs the normal group; ^#^ *P* < 0.05, vs the cold ischaemia at 6 h. I/R, ischaemia‐reperfusion; mTOR, mammalian target of rapamycin; LC‐II, lateral compression type 2; LC3‐II/I, lateral compression type 3‐II/I; LAMP2, lysosome‐associated membrane protein 2; PINK1, PTEN‐induced kinase 1; PIP, peak inspiratory pressure; W/D, wet/dry; TUNEL, terminal deoxynucleotidyl transferase‐mediated dUTP‐biotin nick end labelling. All data are expressed as mean ± SD; comparisons among multiple groups are analysed by the one‐way ANOVA; all experiments are repeated three times

Next, all rats were treated with I/R at different time intervals in order to evaluate the influence of this treatment on lung tissue injury, mitochondrial autophagy and cell apoptosis. Immunofluorescence LC‐II/LAMP2 double staining and transmission electron microscope were employed to detect mitochondrial autophagy in donor lungs. Compared with the normal group, fluorescence intensity of mitochondrial autophagy markers LC‐II and LAMP2 was significantly higher at each time interval, and mitochondrial autophagy gradually increased, and even attained peak values after 6 hours (Figure 2A) (all *P* < 0.05). After performing cold ischaemia preservation for 6 hours, the levels of mitochondrial autophagy‐related proteins (LC3‐II/I, Beclin‐1, PINK1 and Parkin) were found to be elevated, which reached peak levels at 6 hours, and gradually decreased from 6th to 12th hours. However, the expression of p‐mTOR was suppressed, and p‐mTOR/mTOR expression was also reduced, which reached the lowest point at 6 hours, and then gradually elevated (Figure 2B) (all *P* < 0.05). The above results suggested that autophagy inhibited the mTOR pathway. In addition, TUNEL staining was performed to detect cell apoptosis in all groups. After reperfusion, the proportion of apoptotic cells was markedly increased, and reached a peak value after 6 hours (Figure 2C) (all *P* < 0.05). The W/D ratio and PIP values of lung tissues in each group were found to be significantly elevated, while PO_2_ was reduced, and reached peak values after 6 hours (Figure 2D‐F) (all *P* < 0.05). The above findings demonstrated that after I/R of donor lung in rat models of orthotopic lung transplantation, mitochondrial autophagy evidently increased, reached peak values after 6 hours, and then gradually decreased. The elevation of mitochondrial autophagy was accompanied by promoted cell apoptosis, aggravated lung injury and impaired lung functioning.

### 3.2 Inhibition of mitochondrial autophagy reduces while inhibition of mTOR pathway aggravates lung injury after I/R of donor lung

**Figure 3:**
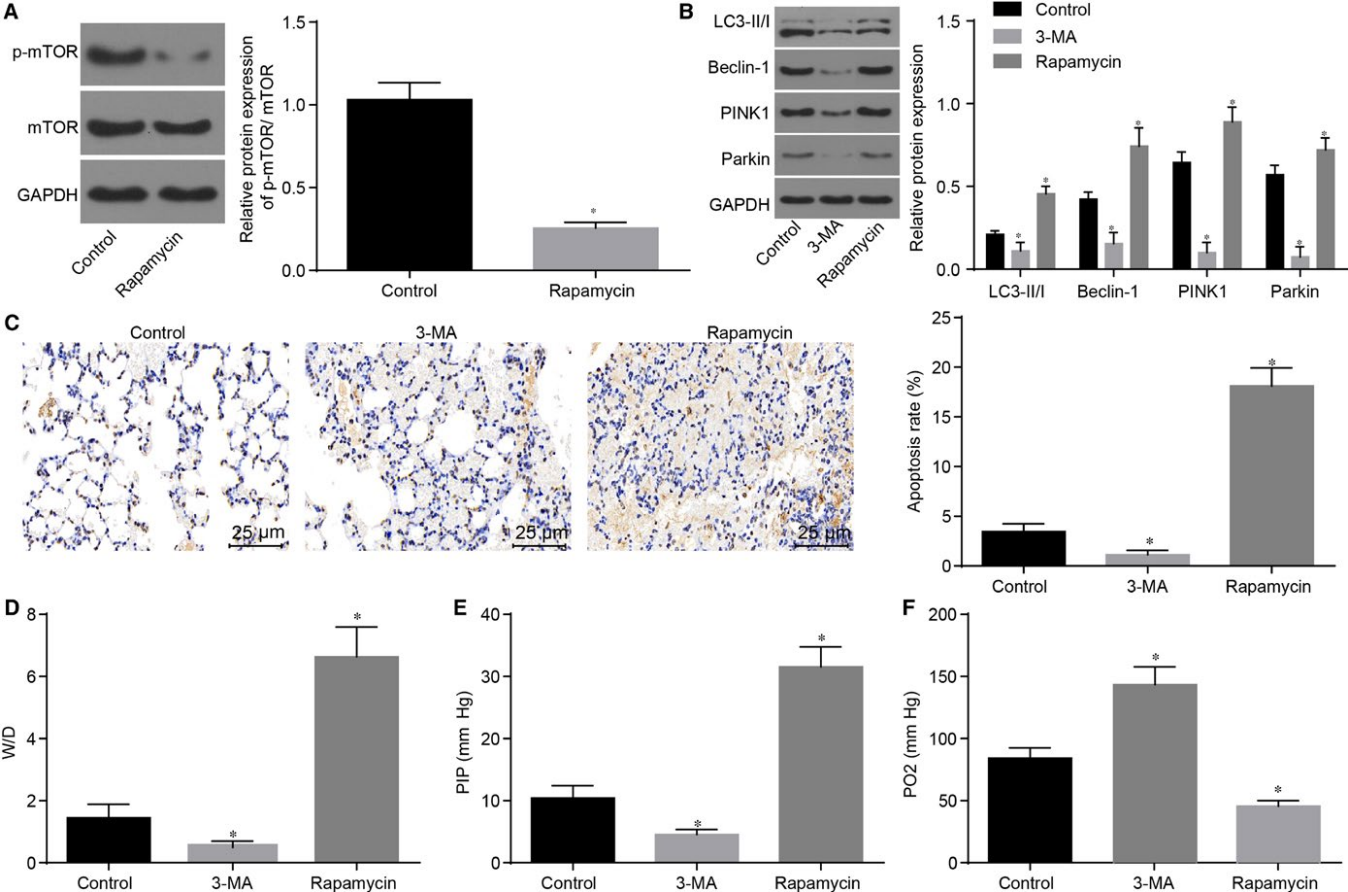
Repression of mitochondrial autophagy alleviates while inhibition of the mTOR pathway worsens lung injury after I/R of donor lung. A, Western blot analysis shows that Rapamycin inhibits the protein expression of p‐mTOR/mTOR; B, Western blot analysis shows that 3‐MA evidently reduces protein levels of LC3‐ II/I, Beclin‐1, PINK1 and Parkin; C, TUNEL staining (× 400) indicates that 3‐MA reduces cell apoptotic rate; D, 3‐MA reduces W/D ratio; E, 3‐MA reduces PIP value; F, 3‐MA elevates PO_2_ value; *P < 0.05, vs the control group; I/R, ischaemia‐reperfusion; mTOR, mammalian target of rapamycin; LC3‐II/I, lateral compression type 3‐II/I; LAMP2, lysosome‐associated membrane protein 2; PINK1, PTEN‐induced kinase 1; PIP, peak inspiratory pressure; W/D, wet/dry. All data are expressed as mean ± SD; comparisons among multiple groups are analysed by the one‐way ANOVA; all experiments are repeated three times

In order to further explore the intervention of I/R on mitochondrial autophagy and the impact of repression of the mTOR pathway on lung tissue injury, the I/R was intervened at 6 hours and samples were categorized into the control, 3‐MA and Rapamycin groups. Compared with the control group, the Rapamycin group displayed significantly decreased extent of mTOR phosphorylation and p‐mTOR/mTOR expression ratio (*P* < 0.05), suggesting that Rapamycin inhibits the activation of the mTOR pathway. Compared with the control group, the 3‐MA group showed evidently reduced protein levels of LC3‐ II/I, Beclin‐1, PINK1, Parkin (Figure 3A), cell apoptotic rate, (Figure 3B), W/D ratio and PIP value, but significantly elevated PO_2_ values (Figure 3D, E), whereas the Rapamycin group presented with opposite trends (all *P* < 0.05). The above findings suggested that suppression of mitochondrial autophagy via 3‐MA could inhibit cell apoptosis induced by I/R of donor lung in order to alleviate lung injury. However, the inhibition of the mTOR pathway induced the mitochondrial autophagy, thus enhancing the cell apoptosis and aggravating the lung injury.

### 3.3 Mitochondrial autophagy inhibits apoptosis of PMVECs exposed to hypoxia while promoting apoptosis of hypoxia‐exposed PMVECs treated with reoxygenation

**Figure 4:**
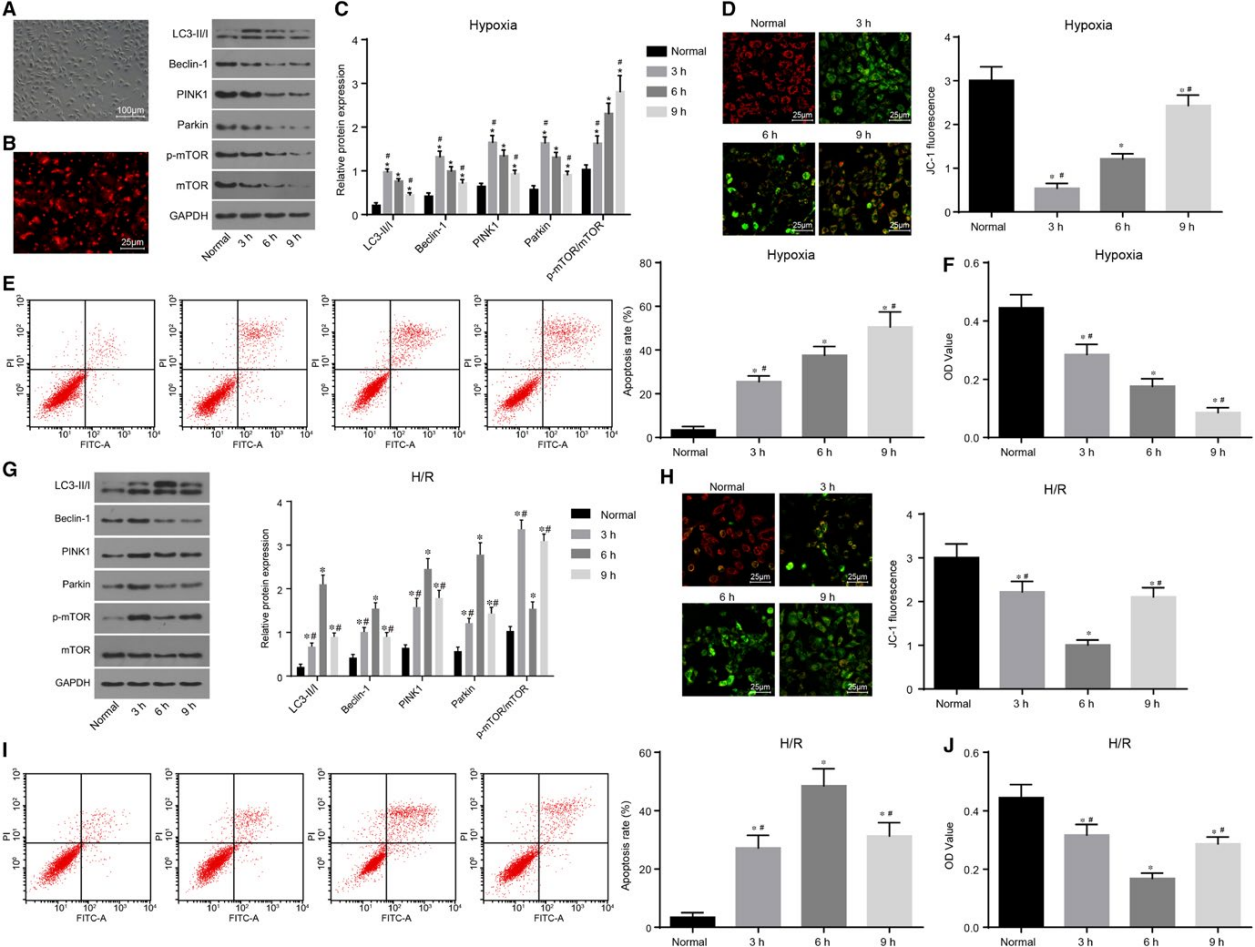
H/R‐induced mitochondrial autophagy promotes apoptosis and suppresses proliferation of PMVECs. A, cell morphology observed under an inverted microscope (× 100); B, CD31 positive expression detected using immunofluorescence (× 200); C and G, protein levels of LC3‐II/I, Beclin‐1, PINK1 and Parkin and the ratio of p‐mTOR/mTOR expression in the hypoxia and H/R groups; D and H, MTP detected by JC‐1 staining in the hypoxia and H/R groups (× 400); E and I, flow cytometric detection of cell apoptotic rate in the hypoxia and H/R groups; F and J, PMVEC proliferation in the hypoxia and H/R groups; *P < 0.05, vs the normal group; ^#^ *P* < 0.05, vs the cold ischaemia at 6 h. H/R, hypoxia‐reoxygenation; PMVECs, pulmonary microvascular endothelial cells; mTOR, mammalian target of rapamycin; LC3‐II/I, lateral compression type 3‐II/I; PINK1, PTEN‐induced kinase 1; MTP, mitochondrial transmembrane potential; OD, optical density; TUNEL, terminal deoxynucleotidyl transferase‐mediated dUTP‐biotin nick end labelling. All data are expressed as mean ± SD; comparisons among multiple groups are analysed by the one‐way ANOVA; all experiments are repeated three times

Cell morphology was observed under an inverted microscope (Figure 4A). Cell morphology was presented with polygonal or shuttle shapes with uniform size, clear boundary and cell nucleus, growing like pave stones or cobblestones. Additionally, immunofluorescence was employed to detect the expression of CD31 (Figure 4B) in all groups. These results indicated that the isolated cells were PMVECs.

The PMVECs were randomly divided into the hypoxia and H/R groups and observed. Protein levels of LC3‐II/I, Beclin‐1, PINK1 and Parkin increased initially and then decreased. Under hypoxic conditions, mTOR was activated, and mitochondrial autophagy was inhibited, and the expression ratio p‐mTOR/mTOR was elevated (Figure 4C), and MTP decreased initially and then increased (Figure 4D) in the hypoxia group. In addition, mitochondrial autophagy induced by hypoxia antagonized cell apoptosis. At first, with derivation, mitochondrial autophagy exerted a protective effect on PMVECs against apoptosis. As mitochondrial autophagy tended to attain normal levels, cell apoptosis was promoted (Figure 4E). The H/R group displayed evidently increased protein levels of LC3‐II/I, Beclin‐1, PINK1 and Parkin, down‐regulated extent of mTOR phosphorylation and p‐mTOR/mTOR expression ratio with elevated mitochondrial autophagy (Figure 4G), and reduced MTP, which attained peak values after 6 hours (Figure 4H). Mitochondrial autophagy induced by H/R promoted apoptosis and suppressed cell proliferation (Figure 4 E, F, I, J).

**Figure 5:**
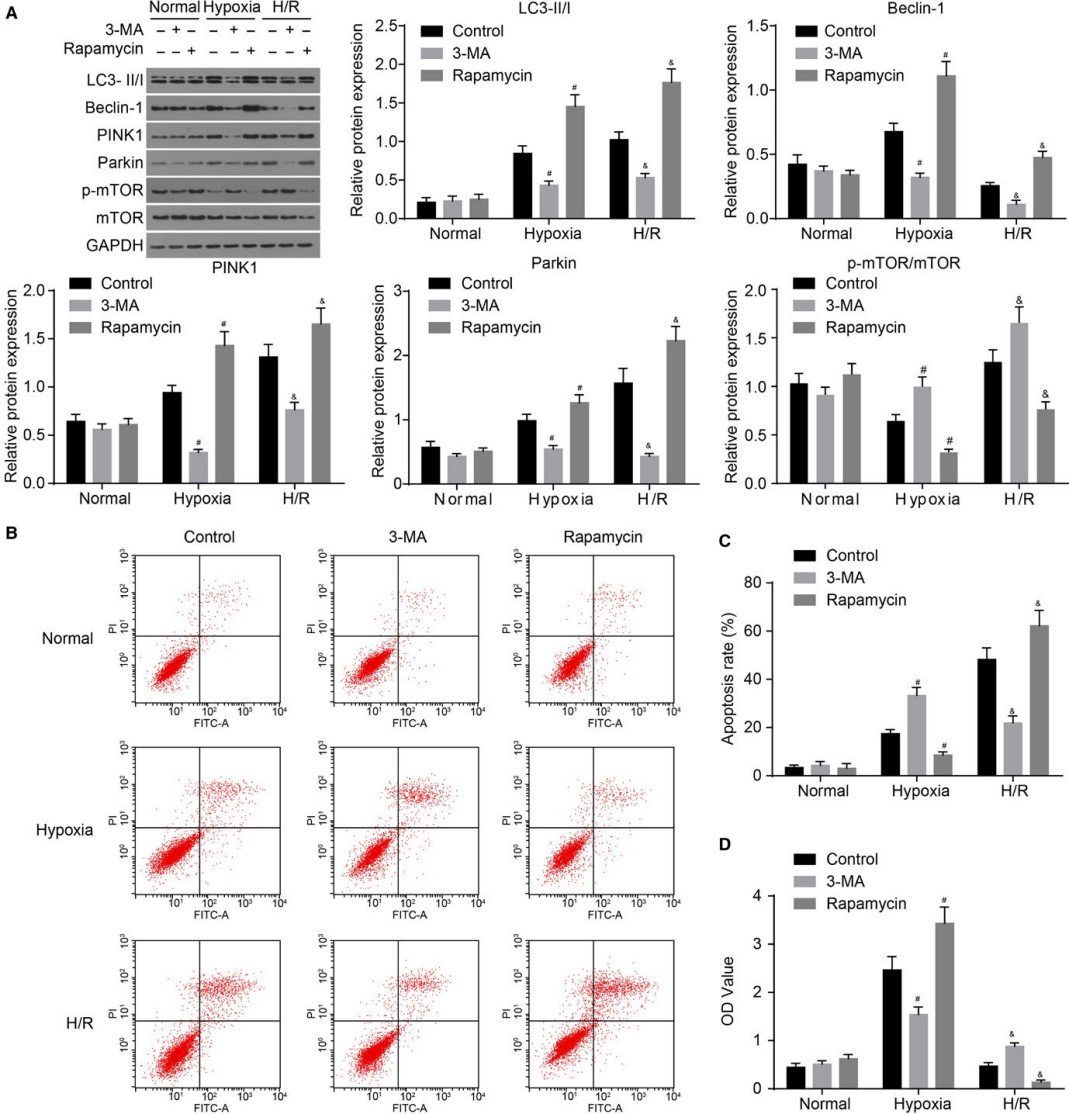
3‐MA inhibits H/R‐induced mitochondrial autophagy to inhibit apoptosis and promote proliferation of PMVECs. A, protein bands and protein levels of LC3‐II/I, Beclin‐1, PINK1, Parkin and mTOR, and the extent of mTOR phosphorylation in hypoxia‐ and H/R‐exposed PMVECs treated with 3‐MA or Rapamycin; B, flow cytometric detection for apoptosis of hypoxia‐ and H/R‐exposed PMVECs treated with 3‐MA or Rapamycin; C, PMVEC apoptosis after treatment of 3‐MA or Rapamycin in the process of PMVECs treated with hypoxia and H/R; D, PMVEC proliferation after treatment of 3‐MA or Rapamycin in the process of PMVECs treated with hypoxia and H/R; *P < 0.05, vs the normal group; ^#^ *P* < 0.05, vs the hypoxia +control group; ^&^ *P* < 0.05, vs the H/R+ control; H/R, hypoxia‐reoxygenation; PMVECs, pulmonary microvascular endothelial cells; mTOR, mammalian target of rapamycin; LC3‐II/I, lateral compression type 3‐II/I; PINK1, PTEN‐induced kinase 1; OD, optical density. All data are expressed as mean ± SD; comparisons among multiple groups are analysed by the one‐way ANOVA; all experiments are repeated three times

At last, the PMVECs exposed to hypoxia for 6 hours and reoxygenation for 3 hours were treated with 3‐MA to inhibit mitochondrial autophagy. It was found that compared with the normal group, the p‐mTOR/mTOR expression ratio increased in the hypoxia group, and was further elevated in the H/R group. After intervention using Rapamycin, a decrease in the p‐mTOR/mTOR expression ratio was noted. Compared with the normal group, protein levels of LC3‐II/I, Beclin‐1, PINK1 and Parkin in PMVECs were found to be evidently increased in the hypoxia group (all *P < *0.05). After reoxygenation treatment, protein levels of LC3‐II/I, Beclin‐1, PINK1 and Parkin were additionally elevated. The mitochondrial autophagy inhibitor 3‐MA exerted no effects on the protein levels of LC3‐II/I, Beclin‐1, PINK1 and Parkin in PMVECs in the normal group, while down‐regulating the protein levels of LC3‐II/I, Beclin‐1, PINK1 and Parkin in PMVECs in the hypoxia and H/R groups (Figure 5A). In the process of hypoxia, mitochondrial autophagy antagonized apoptosis of PMVECs. Apoptosis of PMVECs was found to be promoted after intervention using 3‐MA, while cell proliferation was inhibited. Mitochondrial autophagy was induced by H/R‐mediated apoptosis. After intervention using 3‐MA and down‐regulation of Beclin‐1, cell apoptosis was found to be decreased, while proliferation increased (Figure 5B‐D). The above findings indicated that mitochondrial autophagy exerted anti‐apoptotic effects on PMVECs exposed to hypoxia, while enhancing apoptosis in the hypoxia‐exposed PMVECs treated with reoxygenation.

## 4 DISCUSSION

Lung I/R injury is regarded as the leading cause of primary graft failure after lung transplantation, contributing to significant morbidity and mortality.^22^ In recent years, much focus has been directed to the correlation of mitochondrial autophagy with organ systems and the development of some pulmonary diseases, while the specific mechanisms of mitochondrial autophagy in lung I/R injury remain to be largely unknown.^3^, ^17^ Therefore, the current study was designed and performed with the hypothesis that mitochondrial autophagy could promote I/R‐induced injury by inactivating the mTOR pathway.

Initially, increased protein levels of LC3‐II/I, Beclin‐1, PINK1 and Parkin were detected in lung tissues after I/R, and attained peak values after 6 hours, in addition to elevated apoptosis rate, W/D ratio, PIP value and reduced PO_2_, suggesting that I/R induces mitochondrial autophagy and promotes lung injury. The translocation of LC3‐II, which was achieved by phosphatidylethanolamine conjugation of LC3‐I to the autophagosome membrane was revealed to play a central role in autophagosome formation.^11^ In addition, a previous study reported that up‐regulation of LC3‐II/I and LC3‐II is related to the activation of mitochondrial autophagy.^23^ Beclin‐1, the mammalian orthologue of yeast autophagy‐related protein, is essential for autophagy and modulates mitochondrial autophagy and cell death.^24^, ^25^ Moreover, it was reported that PINK1 in conjunction with Parkin could affect mitochondrial integrity in some tissues, such as dopaminergic neurons.^26^ Parkin is selectively secreted from the cytosol to injured mitochondria so as to stimulate mitochondrial autophagy.^27^ Similarly, the PINK1/Parkin‐directed pathway was previously linked to mitochondrial damage, ubiquitylation and mitochondrial autophagy.^28^

In addition, the current study unfounded that H/R‐induced mitochondrial autophagy promotes apoptosis and suppresses proliferation of PMVECs in order to aggravate lung injury, which implied that lung injury could be attenuated via reduction of mitochondrial autophagy. Mitochondrial autophagy appears under H/R conditions, and acts as a regulator for cellular homeostasis by directly normalizing cell death and survival.^29^ Both mitochondrial autophagy and apoptosis are highly regulated biological processes affecting tissue development and diseases, and a study reported that mitochondrial autophagy induced apoptosis in lung cancer cells.^30^, ^31^ Moreover, induction of mitochondrial autophagy was reported to serve as an inhibitor of cell proliferation.^32^ Similarly, accumulating evidence has demonstrated that apoptosis is induced in ALI, and inhibition of apoptosis attenuates lung injury.^33^, ^34^ Furthermore, increasing evidence has shown that lung injury is followed by repression of proliferation.^35^ It is suggested that I/R‐induced lung injury is accompanied by activated mitochondrial autophagy and apoptosis, and thus inhibits mitochondrial autophagy and apoptosis, indicating amelioration of I/R‐induced lung injury.^36^, ^37^ The aforementioned results are consistent with our findings that inhibition of mitochondrial autophagy by 3‐MA attenuated cell apoptosis, thereby alleviating lung I/R injury.

Notably, the findings of the current study implied that mTOR was involved in H/R‐induced mitochondrial autophagy, and inactivation of mTOR promoted lung I/R injury. As a key factor signalling cell growth and enhancing protein translation, mTOR has been previously related to mitochondrial autophagy.^24^ In line with the current data, autophagy was reported to be regulated by mTOR‐dependent pathways.^38^ It was further confirmed that both mTOR and mitochondrial autophagy exerted crucial effects on the pathogenesis of pulmonary diseases, including ALI.^39^ These findings support the evidence that inhibition of mitochondrial autophagy attenuates lung injury via activation of the mTOR pathway during H/R in PMVECs.

## 5 CONCLUSIONS

The current study concludes that mitochondrial autophagy induced by H/R promotes apoptosis and inhibits the proliferation of PMVECs in order to aggravate I/R‐induced lung injury via inactivation of the mTOR pathway. Therefore, mitochondrial autophagy combined with regulation of the mTOR pathway may elucidate the protective mechanisms against I/R‐induced lung injury, with the potential of serving as a prognostic marker for the treatment of lung I/R injury in the future. Therefore, further studies are warranted to adequately define the detailed mechanism by which mitochondrial autophagy and mTOR pathway inhibition interact in the treatment of lung I/R injury.

## CONFLICT OF INTEREST

The authors declare that they have no conflict of interest.
